# Determination of chemical components of the endemic species *Allium turcicum* L. plant extract by LC-MS/MS and evaluation of medicinal potentials

**DOI:** 10.1016/j.heliyon.2024.e27386

**Published:** 2024-03-13

**Authors:** Polat İpek, Mehmet Nuri Atalar, Ayşe Baran, Mehmet Fırat Baran, Mohammad Mehdi Ommati, Musa Karadag, Murat Zor, Aziz Eftekhari, Mehmet Hakkı Alma, Khaled Zoroufchi Benis, Fidan Nuriyeva, Rovshan Khalilov

**Affiliations:** a Department of Physiology, Dicle University, Diyarbakir, Türkiye; b Department of Nutrition and Dietetic, Faculty of Health Sciences, Iğdır University, Iğdır, Türkiye; c Department of Biology, Graduate Education Institute, Mardin Artuklu University, Mardin, Türkiye; d Malatya Turgut Özal University, Malatya, Türkiye; e Department of Food Technology, Vocational School of Technical Sciences, Batman University, Batman, Türkiye; f Henan Key Laboratory of Environmental and Animal Product Safety, College of Animal Science and Technology, Henan University of Science and Technology, Luoyang, Henan, 471000, China; g Research Application Laboratory and Research Center (ALUM), Iğdır University, Iğdır, Turkiye; h Department of Pharmacognosy, Fenerbahçe University, Ataşehir, İstanbul, Türkiye; i Department of Biochemistry, Faculty of Science, Ege University, Izmir 35040, Türkiye; j Department of Biophysics and Biochemistry, Baku State University, Baku, Azerbaijan; k Nanotechnology and Biochemical Toxicology (NBT) center, Azerbaijan State University of Economics (UNEC), Baku AZ1001, Azerbaijan; l Research Center for Pharmaceutical Nanotechnology, Biomedicine Institute, Tabriz University of Medical Sciences, Tabriz 51665118, Iran; m Department of Process Engineering and Applied Science, Dalhousie University, Halifax, NS, Canada; n Department of Computer Science, Faculty of Science, Dokuz Eylul University, Izmir, Türkiye; o Laboratory of Recognition, Identification and Methods of Optimal Solutions, Institute of Control Systems, Baku, Azerbaijan

**Keywords:** Antimicrobial activity, Cytotoxic activity, Allium turcicum, Zuzubak

## Abstract

The *Allium turcicum* L. (Zuzubak) plant as a cultivated vegetable have various health benefits and consumed as a food. Due to the shortcoming evidence in literature and the importance of this plant in folk medicine, in the present study, for the first time, we evaluated the bioactive profile of components (using LC-MS/MS), cytotoxicity, anticancer, antioxidant, and antibacterial prospectives of Zuzubak methanol extract. Reported results show that the extract is rich in bioactive compounds and has anticancer activity with breast cancer cells (MCF-7), human prostate cancer cells (DU-145), and Human osteosarcoma cancer Cell lines of (IC50) in dose dependent manner in the concentration range of 31.25 μg/mL and 2000 μg/mL for 24 and 48 h. Western blotting results determined that the extract significantly suppressed the growth of U2OS, MCF-7, and DU-145 cancer cells by down expression of Ang-1 (angiogenic protein) and Beclin-1 (autophagy protein) and overexpression of Bax (a proapoptotic protein). The oxidative stress indices showed a reduction in RPE-1 and MCF-7 cells and an upsurge in U2OS and DU-145 cells. Additionally, the antimicrobial assay showed suppression of the growth of various pathogenic microorganisms in 4.00–8.00 μg/concentrations of Zuzubak extract using the microdilution method. The phytochemicals identified showed promising anticancer, antioxidant effects, and antimicrobial properties, representing a valuable herbal source for drug development studies.

## 1 Introduction

Nevertheless, with the advancments in phamaceutical industry, the side effects of synthetic drugs are obvious, which led to the need of using natural medicine [[1], [2]]. The discovery of the therapeutic potential of bioactive compounds including flavonoids and phenolic acids derived from herbal flora in studies has brought an important dynamic to clinical applications [3] Flavanoids, which are in the group of polyphenols rich in plant extracts, prevent the damaging effects of ROS from occurring with their antioxidant activities. These compounds in the extract also have a positive effect by protecting low-density lipoprotein from oxidation with their radical-scavenging antioxidant activities [4]. Detailed chemical compound characterization of plant species is required to evaluate these potential bioactivities. For this evaluation, LC-MS/MS is the preferred method in compound analysis because the device data has high sensitivity [5].

With special flavor charecteristics, the genus *Allium* includes numerous species and *Allium* plants are containing of vitamins and minerals [6]. They are also known as a potential source of non-volatile ingredients including phenolic compounds (saponins, triterpenoids flavonoids) and proteins which are responsible for a medicinal effects (Mnayer Fabiano-Tixier et al., 2014). Epidemiological investigations showeed that the risk of different type of cancer (stomach, colon and liver) incidence could be significantly reduced through the consumption of *Allium* species [7,8]. *Allium* species are tremendously applied for chemopreventive action of organosulfur compounds on colon carcinogenesis and hepatocarcinogenesis. The essential oils of the Allium plants have antimicrobial effects (against various Gram-negative and Grampositive) and antioxidant activities, which are mostly related with their sulfur compounds [8].

One of newly identified endemic member of the Alliaceae family is *Allium turcicum* (Zuzubak) which grows in dry, stony, mixed wooded areas at an altitude of 1152 m in the vicinity of Batman (Sason), Siirt, Halkis Mountain of Turkiye. This plant grows in its natural area in May without any maintenance or special conditions. It resembles a green onion in appearance and local people mostly use in their foods as vegetables, spices, ornamentals, or as medicine for curing different infective and non-infective diseases [[9], [10], [11]].

Due to the shortcoming evidence in literature and the importance of this plant in folk medicine, in the present study, for the first time, we evaluated the bioactive profile of components (using LC-MS/MS), cytotoxicity, anticancer, antioxidant, and antibacterial prospectives of Zuzubak extract.

## 2 Material and method

### 2.1 Collection and extraction of *Allium turcicum*

. In the month of June, Dr. Cumali Keskin was the one who gathered the Allium turcicum from the Batman region. Mardin Artuklu University's herbarium was the location where the plant samples that were collected were evaluated. Allium turcicum, whose taxonomic identity had been previously assessed by Özhatay et al. revealed that its identity has been confirmed. An identification number was assigned to the plant before it was placed in the institution's Herbarium (voucher no. MAU: 2023–26). The plants given in Fig. 1 a and 1 b were collected together with their roots in May 2023 in the Sason district of Batman. The green parts were separated, washed with tap water and distilled water, and dried on plant blotting paper. After drying, 15 g of the plant was weighed and kept in 100 mL of methanol for a week by methanol leaching in room conditions at 25 °C.Then, it was removed from methanol at 70 °C using a Heidolph 94,200 rotary evaporator to obtain the extract in methanol by filtration.

**Fig. 1:**
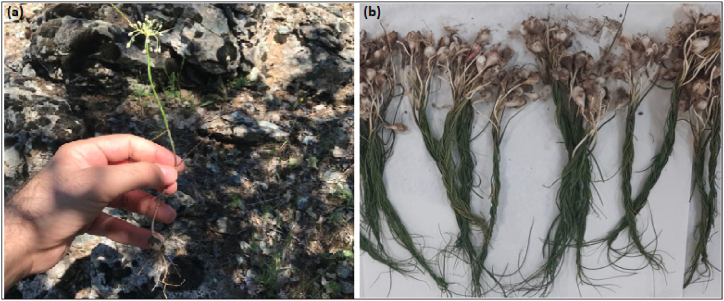
Morphological views of the parts of *Allium turcicum***(a)** after flowering, and **(b)** collected before flowering.

## 3 Determination of phytochemical compound content using LC-MS/MS

### 3.1 Chemicals used and preparation of compound solutions

A liquid chromatography system combined with Triple Quadrupole Mass Spectrometry was used for the qualitative and quantitative analysis of phytochemical compounds. Formic acid, methanol, ammonium formate, water, and hexane are ultra-pure were used. Gallic acid, protocatechuic acid, epigallocatechin, catechin, chlorogenic acid, *4-*Hydroxybenzaldehyde, vanillic acid, caffeic acid, syringic acid, caffeine, vanillin, *p-*coumaric acid, salicylic acid, taxifolin, resveratrol, *trans*-ferulic acid, sinapic acid, scutellarin, *o-*coumaric acid, protocatehuic ethyl ester, rutin, isoquercitrin, hesperidin, quercetin-3-D-xyloside, kaempferol-*3*-glucoside, fisetin, baicalin, *trans*-cinnamic acid, quercetin, naringenin, hesperetin, morin, kaempferol, baicalein, luteolin, biochanin A, and chrysin were obtained high purity. Pure water used for chromatographic purposes was prepared by Millipore, the Milli Q pure water device. All commercially available, high-purity phenolic compounds were used to develop the analytical method. Master main solutions of these compounds with a concentration of 1000 mg/L were prepared. After the dissolution of a solid form of standard chemicals in methanol, the methanol: water (1:1) ratio of the stock solution was filtered using polypropylene housing (0.45 μm pore size and 25 mm diameter) and analyzed using LC-ESI-MS/MS. Compounds were determined by LC-ESI-MS/MS by diluting the stock solution to a 1:1 ratio of methanol and water, respectively.

### 3.2 LC-MS/MS conditions

Qualitative measurement of compounds was performed by High Performance Liquid Chromatography (HPLC) model 1260 Infinity II LC system equipped with a mass spectrometer. A degasser (1260 Degassing), a column furnace (1260 TCC), and dual pumps (1260 Thousand Pumps) were integrated into the reverse phase HPLC device. Conditions were optimized to avoid the effects of adverse conditions such as chromatographic conditions, separation of compounds, and imprinting. In this optimization, a reverse phase Agilent Poroshell 120 EC-C18 model (100 mm x 3.0 mm, 2.7 μm) analytical column was used with chromatographic separation at 25 °C. The elution gradient, solvent flow rate, and injection volume were 0.250 mL min-1 5 μl, 5 mM ammonium formate in water (selective: A) and 0.1% formic acid (selective B) in acetonitrile. The elution gradient was created via the profile (% 10 B (0−1 min), % 40 B (1−3 min), % 70 B (3−5 min), % 40 B (5−6 min), ve 10 % B (6−8 min).

The mass of the compounds were measured by connecting an electrospray ionization (ESI) source, which can operate in negative and positive ionization modes, to the Agilent 6460 Triple Quadruple Mass Spectrometer System model tandem mass spectrometer. Afterwards, LC-ESI-MS/MS data was transferred to Agilent Mass Hunter Software and evaluations were made. The multiple reaction-monitoring (MRM) method was optimized to selectively detect and quantify phytochemical (bioactive) compounds based on screening of ion transitions from the identified precursor phytochemical to the moiety. The collision energies (CE) were controlled and optimized to ensure optimal phytochemical fragmentation and maximum delivery of the desired product ions. MS application conditions were also controlled and made suitable for measurement. The analysis of phenolic compounds by LC-ESI- MS/MS was carried out by the modified method of Atalar et al. A Poroshell 120 EC-C18 (100 mm 4.6 mm ID, 2.7 mm) featured analytical column was used to separate these compounds. As HPLC conditions, the column temperature was set at 40 °C, the injection volume was 5.12 μl, and the flow rate was 0.4 mL/min. Also, the mobile phases including eluent A consisting of 0.1% formic acid and 5 mM ammonium formate in water, and eluent B consisting of 0.1% formic acid, in methanol were used. The elution gradient was applied for two mobile phases. The needle was washed for 3 s in the flush port to minimize the carryover effect before injecting each sample. MS conditions were set as follows: capillary voltage +3.5 kV, nebulizer gas of 35 psi, carrier gas of 8 L/min at 300 °C, and multiple scan modes (negative and positive). Electrospray Ionization (ESI) was used to ionize the samples (Atalar et al., 2021).

### 3.3 Antioxidant activity

A Rel Assay Diagnostic test kit (Cat No: RL0024; Gaziantep, Turkey) was used to measure the total oxidant level **(TOS).**

In this test, the ferrous ion oxidizes the chelator complex to a ferric ion. The oxidation reaction intensity increases due to oxidants in the reaction environment. Ferric ion forms a colored (blue-green) complex in an acidic environment. The colour intensity of the complex, which is proportional to the total amount of oxidant present in the sample, is measured spectrophotometrically at 530 nm wavelength.

For this; 500 μl of reagent-1 and 75 μl of the sample were added to the cell, and the first absorbance point was read at a wavelength of 530 nm. Then, 25 μl of reagent-2 was added to the cell and incubated for 10 min at room temperature, and then the 2nd absorbance point was read at a wavelength of 530 nm.

For calculations the following formules (1), (2) and (3) were used:

(1) $$\text{TOS}:\frac{\left(\Delta \;\text{Absorbance}\;\text{sample}\right)}{\Delta \;\text{Absorbance}\;\text{standard}}\;X\;\text{Standart}\;\text{Value}$$

(2) **Δ Absorbance sample** = 2nd absorbance sample − 1st absorbance sample,

(3) **Δ Absorbance standard** = 2nd absorbance standard − 1st absorbance standard, **Standard value:** 20 μmol H_2_O_2_ equiv./L.

A Rel Assay Diagnostic test kit (Cat No: RL0017; Gaziantep, Turkey) was used to measure the total antioxidant level (TAS).

The principle of the test is based on the oxidation of ferrous ions to ferric ions. Glycerol molecules enhance the oxidation reaction in the reaction medium. In an acidic environment, iron ions form a colored complex with xylenol orange. The intensity of the color depends on the total amount of oxidant molecules. Hydrogen peroxidase is used to calibrate the test.

500 μl reagent-1 was put into the cell, and 30 μl sample was added. For the first absorbance point, it was read at a wavelength of 660 nm. Then, 75 μl reagent-2 was added to the cell, incubated for 10 min at room temperature, and read “2nd time" at a wavelength of 660 nm.

Calculation of Results (4):

(4) TAS (mmol/L) = (Δ Abs Std1- Δ Abs Sample) / (Δ Abs Std1- Δ Abs Std2)

Δ Abs Std1: 2nd absorbance value of Std1-1st absorbance value of Std1
Δ Abs Std2: 2nd absorbance value of Std2 - 1st absorbance value of Std2
Δ Abs sample: 2nd absorbance value of Abs sample - 1st absorbance value of Abs sample

The oxidative stress index (OSI) (5) is an indicator of the degree of oxidative stress and was calculated by dividing the total oxidant level by the total antioxidant level and multiplying the resulting value by 10 [[12], [13], [14]].

(5) $$\text{Oxidative}\;\text{Stress}\;\text{Index}\;\left(\text{OSI}\right)=\frac{\text{Total}\;\text{Oxidant}\;\text{Status}\;\left(\text{TOS}\right)}{\text{Total}\;\text{Antioxidant}\;\text{Status}\;\left(\text{TAS}\right)}*10$$

### 3.4 Suppressing effects of *Allium turcicum* extract on the growth of pathogenic strains

The growth-suppressive effects of Allium turcicum extract on pathogenic strains were tested using the microdilution technique [15]. *Staphylococcus aureus* ATCC 29213 (SA), *Klepiella pneömenia* ATCC 13883 (KP), *Escherichia coli* ATCC 25922 (EC), *Pseudomonas aeruginosa* ATCC 27833 (PA), *Listeria monocytogenes* ATTC 7644 and *Candida albicans* (CA) Microorganisms were used in experimental studies.

Each microorganism was grown in appropriate nutrient media and a solution of each strain was prepared according to 0.5 Mcfarland turbidity standard (1.5 × 108, CFU/mL) [16]. Müller hilton broth and RPM 1640 media were prepared and distributed on microplates for antimicrobial effect applications. The plant extract was prepared in varying concentrations and microdilution was performed starting from the first well. Microorganisms prepared according to Mcfarland 0.5 turbidity standard were pipetted into the extract medium in varying concentrations in the appropriate medium. Comparisons were made in terms of antimicrobial effect by applying the appropriate antibiotic for each strain. Each microorganism was incubated for 24–48 h in an oven at the optimum temperature (25–37 °C). At the end of the period, the microplates were checked for MIC.

### 3.5 Viability suppressing effects of *Allium turcicum* extract on cancer cells

The cytotoxic effect of Allium turcicum extract on cell lines was conducted at Dicle University Faculty of Veterinary Medicine Cell culture laboratories using the MTT assay method. One healthy cell line and different cancer cell lines were used in practice to examine the suppression of percent viability due to the cytotoxic effect. Retinal pigment epithelium (RPE-1) cell lines were used as healthy cell lines, and Human Osteosarcoma (U2OS), human breast adenocarcinoma (MCF-7), human prostate cancer (DU-145) cell lines were used as cancer cells. RPMI-1640 (Sigma-Aldrich R8758, USA) was used for the culture of cell lines in T75 culture flasks. Antibiotics (streptomycin and penicillin) were added to the cultured medium at a rate of 10% FBS and 100 U/mL. Cells were incubated at 37 °C in a 5% CO_2_ environment with 80–90% confluency. Cells were taken from flasks with trypsin and counted by the hemocytometric method. Each cell line was then cultured in 96-well microplates. The extract prepared at different concentrations was added to the culture medium in which the cells were found, and interaction was ensured for 24 and 48 h. Some wells of microplates were used for control steps. To evaluate the changes in cell viability after the interaction period, the MTT test (Merck) at a wavelength of 540 nm using a plate reader (Multi Scan Go, Themo) was performed in a dark environment. Using the data obtained as a result of the test, the concentration (IC50) values at which the cells showed 50% viability for the extract were calculated using the GraphPad Prism. Results were analyzed with the IBM SPSS 21.0 program and the significance level was evaluated as p < 0.05 [,^17^].

### 3.6 Western blot

RPE-1, U2OS, MCF-7, and DU-145 cells were incubated at 1x106 in cell culture flasks for one day. The next day, 24 h of Alium turcicum was applied to the cells at effective doses determined in MTT, and interaction was ensured. Ultra-poor water was applied to the control group. Cell lysates were prepared using Radioimmunoprecipitation assay buffer (RIPA) lysis buffer containing protease-phosphatase inhibitor after administration. Total cellular protein concentration was adjusted to 20 μg/mL by measuring using a Branch Chain Amino Acid (BCA) protein assay kit according to the manufacturer's instructions (Pierce, Thermo Scientific). For loading the gels, 2x Laemmli sample buffer (containing 5% *p*-mercaptoethanol) was mixed at a ratio of 1:1–20 μg of total protein and heated at 95 °C for 5 min. Next, the samples were electrophoresed (protein separation) on a sodium dodecyl sulfate gel transferred to a Polyvinylidene fluoride membrane, and then blocked for 1 h in PBS-T containing 5% skim milk. Incubation of blocked membranes was performed with primary antibodies (Beclin-1, Bax, Ang-1, and β-actin as internal control) diluted 1:1000 overnight at 4 °C. The next day, the membranes were washed and incubated for 1 h at room temperature with a 1:1000 ratio of secondary antibodies. In the final step, antibodies were washed with PBST, bands were visualized with an ECL kit and a ChemiDoc™ MP imaging system (Bio-Rad Laboratories, Inc., California, USA) and measured with Image-j software [18].

## 4 Results and discussion

### 4.1 LC-MS/MS data *of Allium turcicum* extract

The compound profile of *Allium turcicum* extract in terms of phytochemicals was evaluated with the findings obtained from LC-MS/MS data (Fig. 2, Fig. 3). The extract was found to be rich in flavonoid and phenolic acid derivatives. The highest concentration in the profile was rutin (1723.58 μg/g), as well as vanillic acid (96.87 μg/g), *trans*-ferulic acid (84.90 μg/g), isoquercitrin (73.45 μg/g), chlorogenic acid (20.49 μg/g), caffeic acid (28.76 μg/g), resveratrol (26.37 μg/g), vanillin (11.18 μg/g), hesperetin (11.93 μg/g) such as were also found in high amounts to contain bioactive compounds. Compounds such as *trans*-cinnamic acid, kaempferol-3-glucoside, and *p-*coumaric acid were also found to be significant in terms of quantity (Fig. 2 and Table 1).

**Fig. 2:**
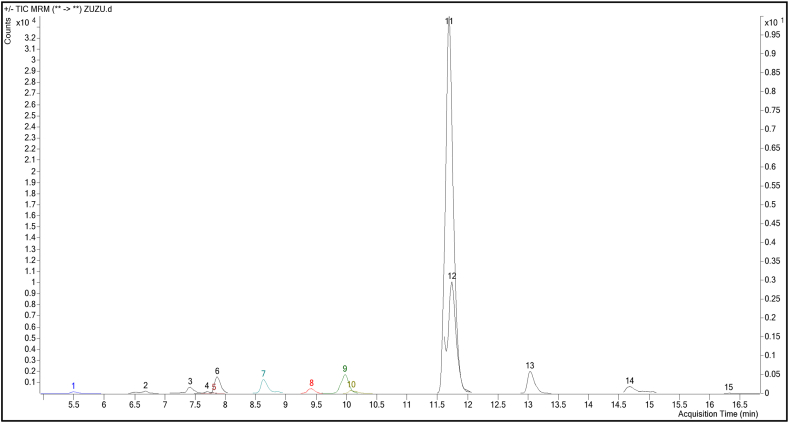
The multiple reaction-monitoring (MRM) chromatograms of *Allium turcicum* leaf extract analyzed by using LC-MS/MS (**1-**Protocatechuic acid, **2-**Epigallocatechin, **3-**Chlorogenic acid, ***4-***Hydroxybenzaldehyde, **5-**Vanillic acid, **6-**Caffeic acid, **7-**Vanillin, **8-***p***-c**oumaric acid, **9-**Resveratrol, **10-***trans*-ferulic acid, **11-**Rutin, **12-**Isoquercitrin, **13-**Kaempferol-3-glucoside, **14-***trans*-cinnamic acid, **15-**Hesperetin).

**Fig. 3:**
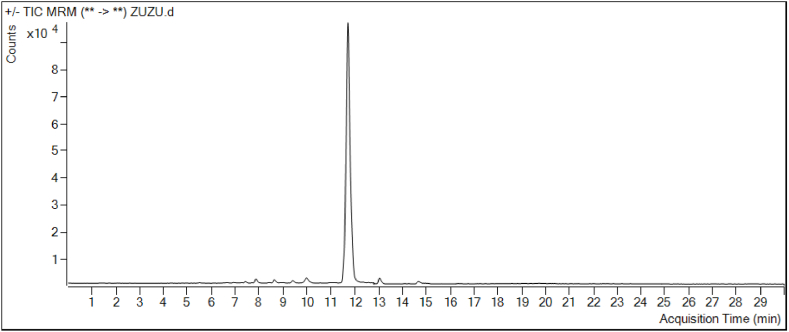
Total Ion Chromatogram (TIC) profile of Allium turcicum leaf extract analyzed by using LC-MS/MS.

**Table 1:** Compounds and their amounts found in the LC-MS/MS profile of *Allium turcicum* leaf extract.

No	Compound	RT	Transition (*m*/*z*)	*Allium turcicum* leaf extract (μg/g)
1	Protocatechuic acid	5.50	153.0 -> 109.0	3.82
2	Epigallocatechin	6.70	307.0 -> 139.0	1.75
3	Chlorogenic acid	7.40	353.0 -> 191.0	20.50
4	*4*-Hydroxybenzaldehyde	7.70	121.0 -> 92.0	3.10
5	Vanillic acid	7.80	167.0 -> 151.8	96.90
6	Caffeic Acid	7.90	178.9 -> 135.1	28.80
7	Vanillin	8.60	153.0 -> 125.0	11.20
8	*p*-coumaric acid	9.40	163.0 -> 119.1	5.60
9	Resveratrol	9.98	229.0 -> 107.0	26.38
10	*trans*-ferulic acid	10.10	193.1 -> 133.9	84.90
11	Rutin	11.70	611.0 -> 302.8	1723.60
12	Isoquercitrin	11.75	464.9 -> 302.8	73.45
13	Kaempferol-3-glucoside	13.03	448.8 -> 286.9	8.13
14	*trans*-cinnamic acid	14.678	149.0 -> 131.1	6.431
15	Hesperetin	16.313	300.9 -> 164.0	11.928

As seen in Figs. 2–3, and Table 1 and it was determined that more than 80% of the extract content in terms of amount belonged to the rutin compound. Surprisingly, rutin was detected at a higher rate in *Allium turcicium* extract. Rutin is a flavonoid, showing bioactivity like a vitamin, making it a very beneficial compound for health. Surprisingly, rutin, which is expected to be abundant in citrus fruits, is abundant in *Allium turcicum* extract, and its high biological activities may increase the importance of this extract.

It is thought that the significant amount of this compound including vanillic acid, *trans*-ferulic acid, chlorogenic acid, and caffeic acid as natural antioxidant compounds in *Allium turcicum* extract will contribute pharmacologically to the mentioned biological activities by being a natural vanillic acid source.

### 4.2 Total oxidant and antioxidant capacity

In Fig. 4 and Table 2, Total oxidant Status (TOS) and total antioxidant (TAS) values of *Allium turcicum* extract were examined on MCF-7, U2OS, DU145, and RPE-1 cells. When the total oxidant Status (TOS) values were calculated, it was observed that there was an increase in the treatment groups in U2OS and DU145 cells in comparison to the control group. A decrease in MCF and RPE cell lines was determined. In addition, while there was a decrease in total antioxidant (TAS) values in the U2OS application group in comparison to the control group, an increase in the DU145, MCF-7, and RPE was determined in compared to the normal group. It is thought that the components contained in Allium turcicium extract provide this effect in the LC-MS profile given in Fig. 2, Fig. 3.

**Fig. 4:**
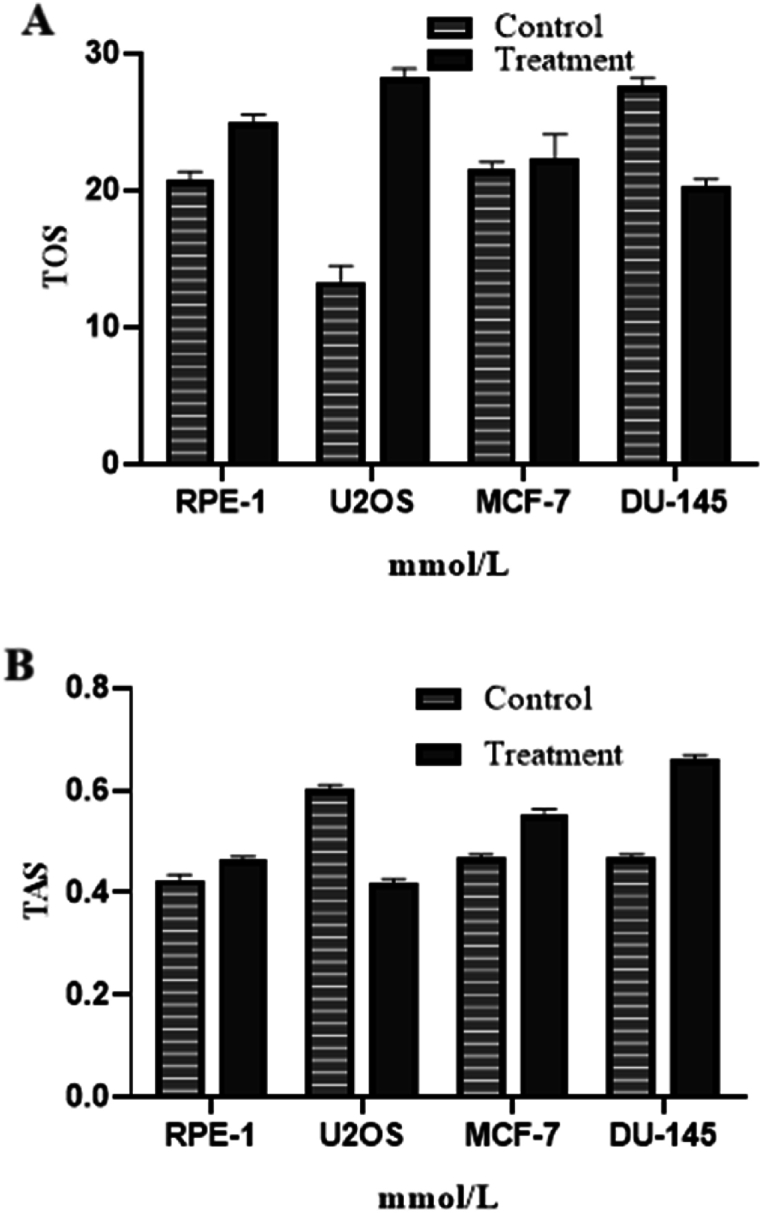
TOS (A) and TAS (B) values based on total oxidant and antioxidant capacity of *Allium turcicum* extract on cell lysates.

**Table 2:** TAS, TOS and OSI results (n = 3, $\bar{X}$ ± S $\bar{x}$, 24 Hours).

	RPE		U20S		MCF		DU145	
	Cont.	Treat.	Cont.	Treat.	Cont.	Treat.	Cont.	Treat.
**TAS**	0.43 ± 0.01	0.46 ± 0.03	0.60 ± 0.01	0.41 ± 0.01*	0.47 ± 0.03	0.54 ± 0.02*	0.47 ± 0.03	0.65 ± 0.06*
**(mmol /L)**								
**TOS**	27.30 ± 0.18	19.57 ± 0.29*	20.09 ± 0.12	24.60 ± 0.43*	21.16 ± 0.20	21.33 ± 0.20	12.51 ± 0.19	27.94 ± 0.07*
**(μmol/L)**								
**OSI**	6.42 ± 0.19	4.24 ± 0.28*	3.34 ± 0.06	6.05 ± 0.25*	4.55 ± 0.38	3.92 ± 0.07*	2.71 ± 0.19	4.30 ± 0.38*

Table X: Data on TAS, TOS and capacity results on cell lysates of *Allium turcicum* extract. p < 0.05. *; The difference between the cell and its control group in the same row is important.

The fact that the occurrence rate in the amount of oxidant substances in the organism (total oxidant capacity) is more than the rate of elimination (total oxidant capacity) is defined as “oxidative stress (oxidative load). The rate of metabolic formation of oxidant substances and the rate of elimination of antioxidants by the antioxidants in a healthy organism in normal conditions away from internal and external adverse conditions is balanced. This situation is called ‘Oxidative equilibrium’. As long as the oxidative equilibrium is maintained, the organism is not damaged by oxidant materials. The ratio of total oxidant levels in organisms to total antioxidant levels gives an Oxidative stress index (OSI). If this index exceeds normal limits, it can damage the organism; as a result, it may cause toxic, pathological, carcinogenic, mutagenic, and degenerative conditions [14].

Besides the increase in antioxidant molecules in response to the increase in oxidative molecules may make the evaluation of TOS measurement alone in terms of oxidative damage misleading. By comparing the change in the TOS/TAS ratio, an accurate comparison is possible by eliminating the antioxidant reactive response that may be misleading for oxidative damage [12].

When the oxidative stress indices of the cell lines we used in our study were compared with the control groups, a decrease in RPE-1 and MCF-7 in the treatment groups, respectively; was determined that there was an increase in U2OS and DU-145. According to these findings, it can be said that Allium turcicum extract treatment prevents oxidative damage in RPE-1 and MCF-7, but not in U2OS and DU-145.

### 4.3 Cytotoxic effect of *Alium turcicum* extract

**Fig. 5:**
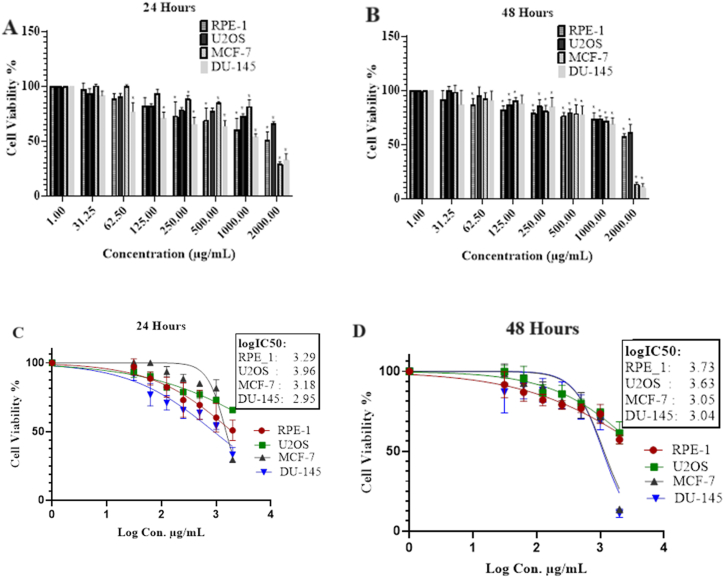
Percent viability rates against varying concentrations in 24 h (A) and 48 h (B) and logIC_50_ values in 24 h (C) and 48-h (D) interactions of *Allium turcicum* extract with healthy and cancer cells. *p < 0.05; The difference between the cell and its control group is important.

In cancer cells, the formation of tubes, adhesion, invasion, and proliferation are all inhibited by compounds from the phenolic and flavonoids families. Not only that, but it also has antiproliferative effects, such as inhibiting the metabolism of neoplastic transformation and chemical carcinogenesis, which it does by having a detrimental effect on the DNA molecule[18]. In this study, percent viability and logIC_50_ rates were dependent on the cytotoxic effects of the active compounds in *Allium turcicum* extract in healthy and cancer cells in the concentration range of 31.25 μg/mL and 2000 μg/mL for 24 and 48 h (Fig. 5 and Table 2). In addition, the 24 and 48-h IC50 values of *allium turcicum* for RPE-1, U2OS, MCF-7, DU-145 cell lines are, respectively; 1964 and 5470 μg/ml; 9257, 4340 μg/ml; 1515, 1144 μg/ml; 906, 1094 μg/ml were found. As a result of the findings, it was discovered that the extract was particularly effective on DU-145 and MCF-7 cancer cells, and it reduced the percentage of viability that these cells possessed. In addition, extract compounds offer a significant inhibitory effect on U2OS cancer cells. Because of their cytotoxic effect, it is believed that certain compounds, including vanillic acid, *trans*-ferulic acid, isoquercitrin, chlorogenic acid, caffeic acid, resveratrol, vanillin, and hesperetin, as well as rutin in the extract, are effective in suppressing vitality [19].

Despite the fact that the same cells are used in studies with extracts from various plant sources, the results of the effects are also different. This is due to the fact that the extracts have different compound profiles. In the course of 48 and 72 h of interaction between *Psidium guajava* L. extract and DU-145 cells, it was determined that high concentrations of compounds present in the extract reduced the viability of the cells by 36.1 and 3.59 %, respectively. A significant contribution to this effect was made by the presence of high levels of polyphenolics (165.60 ± 10.39 mg/g) and flavonoids (82.90 ± 0.22 mg/g) in the structure of the extract. Following the application of the extract to DU-145 cells at concentrations of up to 1.0 mg/mL, it was discovered that it exhibited an antimetastasis effect by inducing caspase-3 action [20]. In a study using *Leea indica* leaf extract on the same cancer cells, it was stated that the IC_50_ value on these cells was 529.44 ± 42.07 μg/mL [21]. In the study of the cytotoxic effect of *Artocarpus heterophyllus* extract on MCF-7 cancer cells, it was concluded that the extract did not have any activity on these cells [22]. In a study conducted with *Marrubium persicum* extract on MCF-7 cells, it was stated that the cytotoxic activity of the extract increased and the cell viability decreased due to the increase in the interaction time of the extract [23]. Their results indicated that extract of *Ephedra foeminea Forssk.* plant suppresses migration and viability on U2OS cancer cells [24]. In another study with U2OS cells, it was concluded that Camellia sinensis extract has apoptotic and viability-suppressing effects [25].

### 4.4 Protein expression of bax

**Fig. 6:**
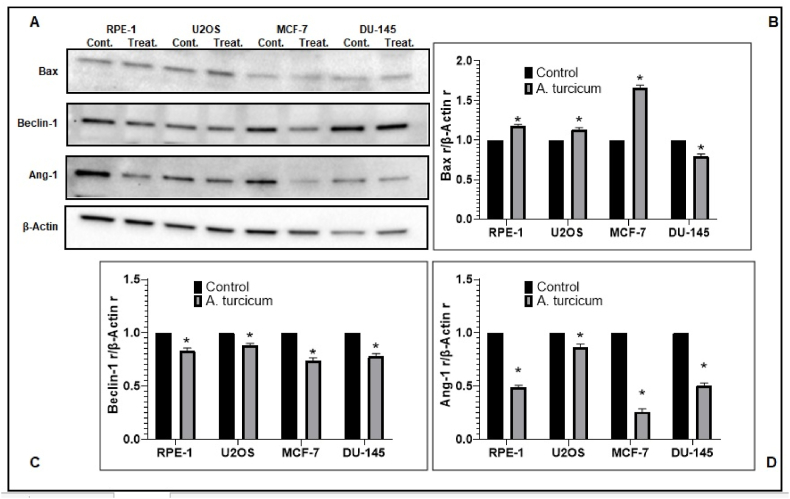
*Allium turcicum* extract; **(a)** Western Blot images, **(b)** Densitometry analysis of Bax density, **(c)** Densitometry analysis of Beclin-1 density, **(d)** Densitometry analysis of Ang-1 density. Western blot analysis showed that Allium turcicum up-regulated Bax protein and down-regulated Beclin-1 and Ang-1. *P < 0.05; The difference between the cell and its control group is important.

In characterizing the cytotoxic effect of *Alium turcicum*, the level of Bax protein expression was evaluated by western blotting, using the data given in Figure-6/A and Figure-6/B. When the results were examined, it was determined that the Bax protein level increased significantly compared to the control groups.

Overexpression of Bax, a proapoptotic protein, induces cytochrome *c* release. Released cytochrome *c* forms a complex in the cytosol with Apaf-1 and the unprocessed caspase-9 proform. Formed in the presence of dATP or ATP, this complex activates key caspases that can trigger a cascade [26]. This increase in the amount of Bax in our study shows that *Allium turcicum* stimulates this apoptotic pathway. As a result of the literature review, no in vitro study was found to determine the amount of Bax as a result of the application of *Allium turcicum* to cancer cells. The data in this study showed that apoptosis induced by *Alium turcicum* administration may be due to overexpression of Bax.

### 4.5 Protein expression of Beclin-1

To characterize the cytotoxic effect of *Alium turcicum*, the level of Beclin-1 protein expression was evaluated by western blotting, and the findings obtained in Fig. 6/a and figure-6/c are given. Results: It was observed that Beclin-1 protein level decreased significantly in *Alium turcicum* applied groups compared to control groups. Autophagy, which plays important roles in both cell death and cell survival, ensures the maintenance of cellular homeostasis by destroying and then recycling damaged cytoplasmic compounds. Beclin-1 is the first autophagy gene shown to be associated with cancer. After the formation of cancer, cancer cells can use autophagy to recover from the stresses of adverse conditions. Thus, autophagy in tumor cells may lead to tumor suppression, leading to increased tumor cell viability [27,]. The tumor suppressor functions of Beclin-1 are mediated by loss of protein expression in cancer cells [28]. As a result of the study using *Allium turcicum* extract, it was determined that the expression of Beclin-1 was weaker in the extract group in comparison to the control groups. It is thought that further studies should also analyze beclin-1 expression in other cancer cell lines to see if the reduced expression of beclin-1 is specific to these cell lines.

### 4.6 Protein expression of Ang-1

In characterizing the cytotoxic effect of *Alium turcicum* extract, the level of Ang-1 protein expression was measured by western blotting. Obtained results are given in figure-6/a and figure-6/d. In the findings, it was observed that Ang-1 protein level decreased significantly in the groups treated with *Alium turcicum* extract compared to the control groups.

Angiogenesis is new vascularization. In most physiological conditions, such as wound healing, angiogenesis is transient, and neovascularization recedes after it has done its job. Angiogenesis primarily involves the stimulation of cell migration and endothelial cell proliferation. Angiogenesis can be a natural physiological event in the human body, or it can be pathological. There are many angiogenic factors for tumor development and metastasis. As key mediator of angiogenesis, Angs regulate the survival of endothelial cells in non-malignant/malignant tissues [29]. Ang-1, as one of the Angs, is the main tyrosine kinase receptor Tie-2 activator, which leads to the autophosphorylation of receptors, and migration of endothelial cells [[28], [30], [31]]. Angiopoeitin-1 has also been demonstrated to act as a critical regulator of embryonic and postnatal neovascularization, although its role in tumor neovascularization (New Vessel Development) is controversial [32]. Also, Angiopoeitin-1 suppresses apoptosis in microvascular endothelial cells, and aortic human umbilical vein endothelial cells (HUVECs) [28,].

Our results showed that Allium turcicum prevents the suppression of apoptosis by causing downregulation of Ang-1 and inhibits angiogenesis. These findings showed that *Allium turcicum* extract induced apoptosis in the cell lines. Therefore, it is thought that *Allium turcicum* can be further investigated as a new alternative chemotherapeutic agent.

### 4.7 Antimicrobial effects of the compounds in the extract

**Fig. 7:**
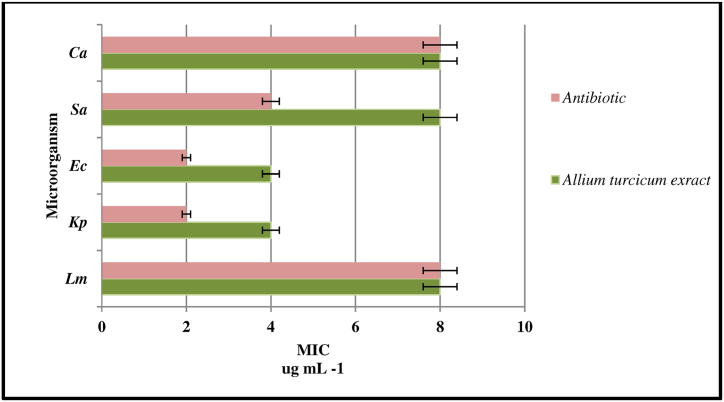
MIC values of *Allium turcicum* extract and antibiotics in suppressing the growth of pathogenic microorganisms.

Microdilution studies of the antimicrobial effect of *Allium turcicum* extract on the suppression of the growth of pathogenic microorganisms were carried out. MIC values with suppressed growth were determined by the findings in Table 3 and Fig. 7. It was observed that the plant extract was effective in the concentration range ranging from 4.00 to 8.00 μg/mL. It was determined that the highest effect on Sa and Ec microorganisms was 4.00 μg/mL concentration. It was observed that the extract was effective on Lm and Ca microorganisms at the same concentration as the antibiotic. The MIC values of the extract, which were effective on Sa, Ec, and Kp, were lower than the antibiotic. However, the naturalness of the *Allium turcicum* extract used and the low cost of obtaining and applying the product are the advantages that make its use as an antimicrobial agent important. It is thought that the antimicrobial effect is due to the compounds with high concentration, such as rutin, which have the high concentration in the LC-MS/MS profile given in Figs. 2 and 3 in the content of *Allium turcicium* extract. In an antimicrobial effect study with *Vigna unguiculata* extract, it was found that MICs on *S. aureus*, *E. coli*, and *C. albicans* were 62.5, 100, and 200 μg/μl, respectively [5]. It was stated that *Pseudocedrela kotschyi* extract was effective in suppressing the growth of *S. aureus* and *E. coli* at concentrations of 0.3–0.7 mg/mL [33]. An antibacterial effect was observed in a nanocomposite that contained the rutin component, according to the findings of a previously published study [34] (see Table 4).

**Table 3:** Percent viability rates as a result of the cytotoxic effects of *Allium turcicum* extract due to its interactions with 24 and 48 h cell lines.

Cytotoxic effects of *Allium turcicum* on cell lines (*n* = 3, $\bar{X}$ ± S $\bar{x}$, 24 Hours)							
	31.25 μg/mL	62.50 μg/mL	125.00 μg/mL	250.00 μg/mL	500.00 μg/mL	1000.00 μg/mL	2000.00 μg/mL
**RPE_1**	97.0 ± 0.08	88.5 ± 0.03	82.4 ± 0.04	73.0 ± 0.06	69.3 ± 0.04	60.2 ± 0.05	50.9 ± 0.03
**U2OS**	93.5 ± 0.06	90.3 ± 0.06	82,0 ± 0.03	78.8 ± 0.02	77.6 ± 0.01	72.6 ± 0.03	65.7 ± 0.02
**MCF-7**	100.4 ± 0.02	100.0 ± 0.03	93.5 ± 0.02	88.5 ± 0.03	85.0 ± 0.02	81.5 ± 0.04	29.6 ± 0.02
**DU-145**	91.4 ± 0.06	76.8 ± 0.03	71.0 ± 0.04	65.6 ± 0.01	63.7 ± 0.00	54.1 ± 0.08	33.0 ± 0.04
Cytotoxic effects of *Allium turcicum* on cell lines (*n* = 3, $\bar{X}$ ± S $\bar{x}$, 48 Hours)							
	**31.25** **μg/mL**	**62.50** **μg/mL**	**125.00** **μg/mL**	**250.00** **μg/mL**	**500.00** **μg/mL**	**1000.00** **μg/mL**	**2000.00** **μg/mL**
**RPE_1**	91.7 ± 0.09	86.8 ± 0.05	82.1 ± 0,04	79.1 ± 0.02	76.9 ± 0.04	73.4 ± 0.08	57.2 ± 0.02
**U2OS**	99.8 ± 0.04	95.4 ± 0.06	86.4 ± 0,02	85.8 ± 0.14	79.7 ± 0.09	74.0 ± 0.09	61.5 ± 0.18
**MCF-7**	99.0 ± 0.08	92.4 ± 0.03	90.1 ± 0,04	81.5 ± 0.02	78.6 ± 0.04	71.6 ± 0.01	13.6 ± 0.02
**DU-145**	87.1 ± 0.16	91.0 ± 0.07	88.0 ± 0,06	85.0 ± 0.07	77.8 ± 0.08	69.0 ± 0.07	11.2 ± 0.04

**Table 4:** MIC values of *Allium turcicum* extract and antibiotics, which are used to suppress the growth of microorganisms, have antimicrobial effects.

Microorganism	*Allium turcicum* extract μg/mL	Antibiotic^a^ μg/mL
*Lm*	8.00	8.00
*Sa*	4.00	2.00
*Ec*	4.00	2.00
*Kp*	8.00	4.00
*Ca*	8.00	8.00

a Three different antibiotics were used: **vancomycin** for Lm and Sa microorganisms, **colistin** for Ec and Kp microorganisms, and **flocanozol** for Ca strains.

Studies conducted to examine the phytochemical contents of plant extracts show that some molecules may play a role in the antimicrobial effect (antibacterial, antifungal, etc.). flavonoids and phenolic acids in the extracts are effective in antimicrobial activity [35] and increase the level of ROS in microorganisms. In addition, these compounds in the extract cause structural and functional degradation of cell membranes, inhibition of important enzymes such as DNA gyrase and protein kinase, as well as dehydratase and type III secretion inactivation mechanisms, causing antimicrobial effects [36,37].

## 5 Conclusion

In this study, for the first time, the bioactive compound content of the endemic species *Allium turcicum* extract was determined using LC-MS/MS. In the profile, compounds such as *trans*-ferulic acid, rutin, vanillic acid, isoquercitrin, chlorogenic acid, caffeic acid, resveratrol, vanillin, and hesperetin were found to be in high concentration. It was determined that the compounds in the extract caused significant suppression of U2OS cancer cells, especially on MCF-7 and DU-145 cancer cells. The anticancer effect due to cytotoxic activity was characterized by evaluating the gene expressions of Bax, beclin-1, and ang-1 proteins. In the evaluation made using the OSI capacities of the compounds, it was seen that they prevented oxidative damage in RPE-1 and MCF-7 cells, but not in U2OS and DU-145 cells. It was observed that the extract compounds (4.00–8.00 μg/mL) were effective in suppressing growth on pathogen strains.

As a result of the anticancer, antimicrobial, and antioxidant effects of the bioactive compounds in *Allium turcicum* extract, this extract may contribute significantly to pharmacological studies with its medicinal potential.

## Funding

This work was supported by the 10.13039/501100015617Research Center for Pharmaceutical Nanotechnology, 10.13039/501100004366Biomedicine Institute of Tabriz University of Medical Sciences, Tabriz, Iran (Grant no: 73,733).

## Data availability statement

Data will be made available on request.

## CRediT authorship contribution statement

**Polat İpek:** Writing – original draft, Writing – review & editing, Conceptualization. **Mehmet Nuri Atalar:** Writing – original draft, Writing – review & editing, Investigation, Conceptualization. **Ayşe Baran:** Writing – original draft, Writing – review & editing, Investigation, Conceptualization. **Mehmet Fırat Baran:** Writing – original draft, Investigation, Formal analysis, Conceptualization. **Mohammad Mehdi Ommati:** Writing – original draft, Writing – review & editing, Investigation, Conceptualization. **Musa Karadag:** Writing – original draft, Writing – review & editing, Resources, Formal analysis, Data curation. **Murat Zor:** Writing – original draft, Writing – review & editing, Formal analysis, Data curation, Conceptualization. **Aziz Eftekhari:** Writing – original draft, Writing – review & editing, Project administration, Investigation, Conceptualization. **Mehmet Hakkı Alma:** Writing – original draft, Writing – review & editing, Investigation, Conceptualization. **Khaled Zoroufchi Benis:** Writing – original draft, Writing – review & editing, Visualization, Software, Investigation. **Fidan Nuriyeva:** Visualization, Validation, Software, Formal analysis, Data curation. **Rovshan Khalilov:** Writing – original draft, Writing – review & editing, Visualization, Resources, Conceptualization.

## Declaration of competing interest

The authors declare that they have no known competing financial interests or personal relationships that could have appeared to influence the work reported in this paper.

## Figures and Tables

**figs1:**
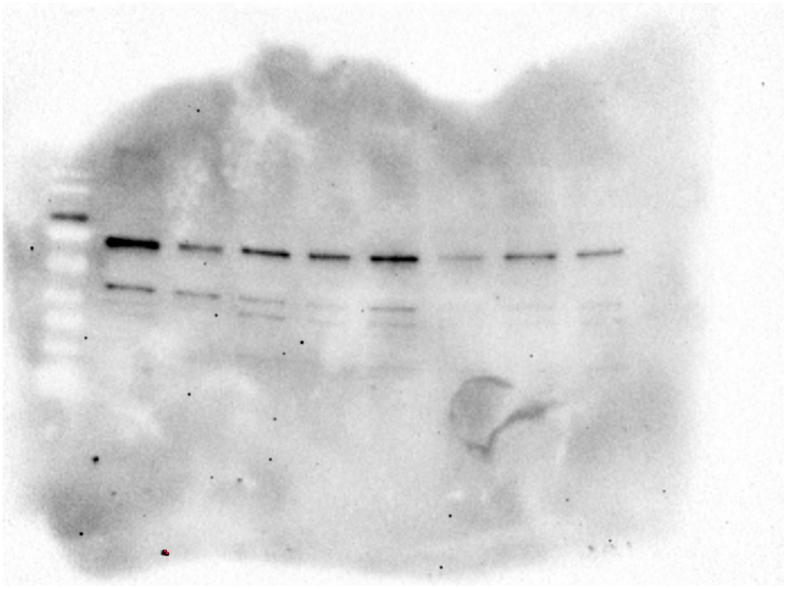


**figs2:**
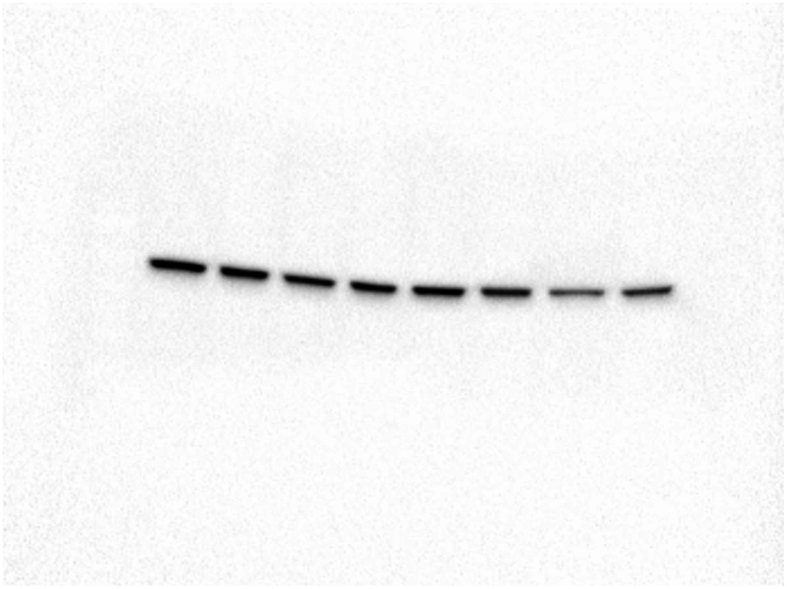


**figs3:**
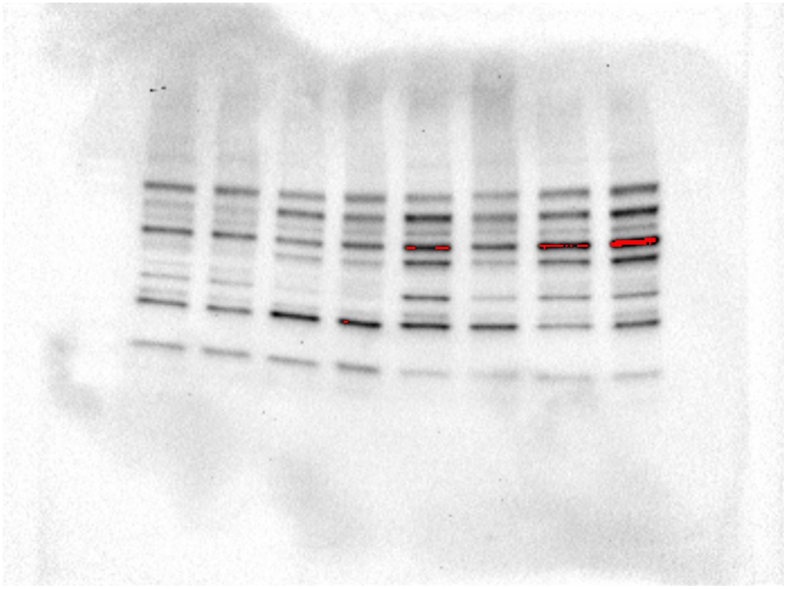


**figs4:**
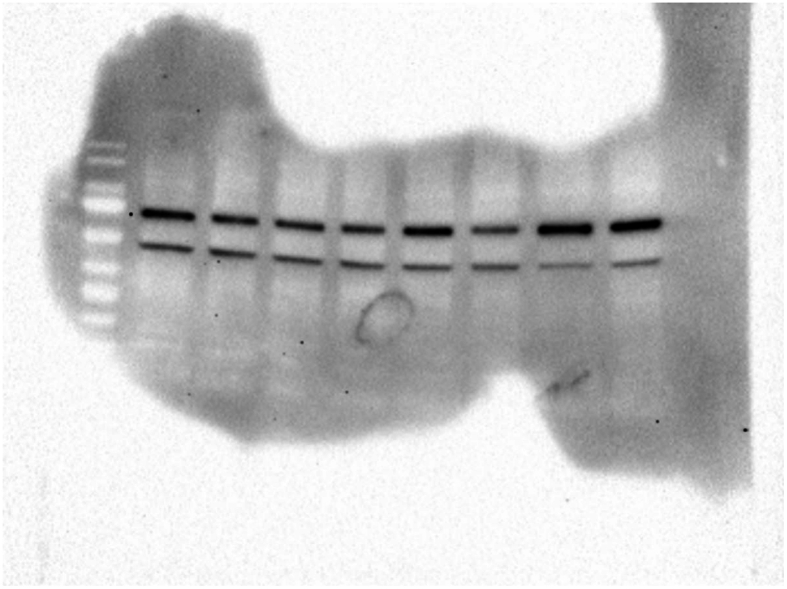


## References

[1] Bouziane T., Daouia H., Soumia A. (2022). In vitro antifungal activity of the extracts of Punica granatum L obtained by reflux method against Fusarium oxysoprum albedenis in south west of Algeria. Advances in Biology & Earth Sciences.

[2] Cherifa A., Nacira G., Haouaria H., Lakhdar M., Ali B., Larbi B. (2022). In Vitro Antifungal Effect Of Cotula Cinerea Extracts Against Fusarium Oxysporum F. Sp. Albedinis And Soil Population Assay. Advances in Biology & Earth Sciences.

[3] Kim TH, Heo SY, Chandika P, Kim YM, Kim HW, Kang HW, Je JY, Qian ZJ, Kim N, Jung WK. (2024). A literature review of bioactive substances for the treatment of periodontitis: In vitro, in vivo and clinical studies. Heliyon.

[4] Fuhrman B., Buch S., Vaya J., Belinky P.A., Coleman R., Hayek T., Aviram M. (1997). Licorice extract and its major polyphenol glabridin protect low-density lipoprotein against lipid peroxidation: in vitro and ex vivo studies in humans and in atherosclerotic apolipoprotein E-deficient mice. Am. J. Clin. Nutr..

[5] Dinore J.M., Patil H.S., Dobhal B.S., Farooqui M. (2022). Phytochemical analysis by GC-MS, LC-MS complementary approaches and antimicrobial activity investigation of Vigna unguiculata (L.) Walp. leaves. Nat. Prod. Res..

[6] Hafez Ghoran S., Rahimi H., Kazemi A., Scognamiglio M., Naderian M., Iraji A., Bordbar F. (2021). Allium hooshidaryae (Alliaceae); Chemical compositions, biological and ethnomedicine uses. J. Ethnopharmacol..

[7] Fleischauer A.T., Poole C., Arab L. (2000). Garlic consumption and cancer prevention: Meta-analyses of colorectal and stomach cancers. Am. J. Clin. Nutr..

[8] Mnayer D., Fabiano-Tixier A.S., Petitcolas E., Hamieh T., Nehme N., Ferrant C., Fernandez X., Chemat F. (2014). Chemical composition, antibacterial and antioxidant activities of six essentials oils from the Alliaceae family. Molecules.

[9] Yousaf Z., Umer A., Younas A., Khan F., Wang Y. (2012). Allelopathic plants: 24. genus Allium L. Allelopathy J..

[10] Cowley N., Jill Özhatay, Mathew B. (1994). New species of Alliaceae & Hyacinthaceae from Turkey. plants People Possiblties.

[11] Koyuncu A. (1997).

[12] Ozsahin Delibas E.A., Kiray E. (2023). Investigation of antioxidant and antimicrobial activities of walnut (Juglans regia L.) kernel septum. The European Research Journal.

[13] Savaş H.B. (2014).

[14] Öztoprak F.S., Yiğitalp Rençber S., Ceylan A., Arıca E., Sayın İpek D.N., Kurt M.E., Yetiz P. (2022). Assessment of lead, mercury, cadmium, chromium and total antioxidant capacity levels of employees exposed to exhaust gases in closed parking lots. Int. J. Environ. Anal. Chem..

[15] Xu Jiajun, Mahmut Yıldıztekin, Han Dayong, Cumali Keskin, Ayşe Baran, Mehmet Fırat Baran, Aziz Eftekhari (2023). Biosynthesis, characterization, and investigation of antimicrobial and cytotoxic activities of silver nanoparticles using Solanum tuberosum peel aqueous extract. Heliyon.

[16] Ganesan R.M., Jeyakanthan J., Gurumallesh Prabu H., Ravikumar S., Poorani G., Boomi P. (2019). Biological synergy of greener gold nanoparticles by using Coleus aromaticus leaf extract. Mater. Sci. Eng. C.

[17] Baran M.F., Keskin C., Baran A., Kurt K., İpek P., Eftekhari A., Khalilov R., Fridunbayov I., Cho W.C. (2023).

[18] Irtegun P. (2018). Sevgi and İpek, Antiproliferative effect of Potentilla fulgens on glioblastoma cancer cells through downregulation of Akt/mTOR signaling pathway. J. Cancer Res. Therapeut..

[19] Gullón B., Lú-Chau T.A., Moreira M.T., Lema J.M., Eibes G. (2017). Rutin: a review on extraction, identification and purification methods, biological activities and approaches to enhance its bioavailability. Trends Food Sci. Technol..

[20] Chen K.C., Hsieh C.L., Peng C.C., Hsieh-Li H.M., Chiang H.S., Huang K.D., Peng R.Y. (2007). Brain derived metastatic prostate cancer DU-145 cells are effectively inhibited in vitro by guava (Psidium gujava L.) leaf extracts. Nutr. Cancer.

[21] Ghagane S.C., Puranik S.I., Kumbar V.M., Nerli R.B., Jalalpure S.S., Hiremath M.B., Neelagund S., Aladakatti R. (2017). In vitro antioxidant and anticancer activity of Leea indica leaf extracts on human prostate cancer cell lines. Integrative Medicine Research.

[22] Patel R.M., Patel S.K. (2011). Cytotoxic activity of methanolic extract of artocarpus heterophyllus against a549, hela and mcf-7 cell lines. J. Appl. Pharmaceut. Sci..

[23] Hamedeyazdan S., Fathiazad F., Sharifi S., Nazemiyeh H. (2012). Antiproliferative activity of marrubium persicum extract in the MCF-7 human breast cancer cell line. Asian Pac. J. Cancer Prev. APJCP.

[24] Mpingirika E.Z., El Hosseiny A., Bakheit S.M.S., Arafeh R., Amleh A. (2020). Potential anticancer activity of Crude Ethanol, ethyl Acetate, and water extracts of Ephedra foeminea on human osteosarcoma U2OS cell viability and migration. BioMed Res. Int..

[25] Er S., Dikmen M. (2017). Camellia sinensis increased apoptosis on U2OS osteosarcoma cells and wound healing potential on NIH3T3 fibroblast cells. Cytotechnology.

[26] Zhou X.M., Chun B., Wong Y., Fan X.M., Zhang H.B., Chia M., Lin M., Kung H.F., Fan D.M., Lam S.K., Min X. (2001). Non-steroidal anti-inflammatory drugs induce apoptosis in gastric cancer cells through up-regulation of bax and bak Protein expressions were determined by western blotting . induced apoptosis in both cells . AGS cells were more. Carcinogenesis.

[27] Liu P.F., Hu Y.C., Kang B.H., Tseng Y.K., Wu P.C., Liang C.C., Hou Y.Y., Fu T.Y., Liou H.H., Hsieh I.C., Ger L.P., Shu C.W. (2017). Expression levels of cleaved caspase-3 and caspase-3 in tumorigenesis and prognosis of oral tongue squamous cell carcinoma. PLoS One.

[28] Brindle N.P.J., Saharinen P., Alitalo K. (2006). Signaling and functions of angiopoietin-1 in vascular protection. Circ. Res..

[29] Coşkun G., Özgür H. (2011). Apoptoz ve Nekrozun Moleküler Mekanizması. Ars.

[30] Wang J., Wu K., Zhang D., Tang H., Xie H., Hong L., Pan Y., Lan M., Hu S., Ning X., Fan D. (2005). Expressions and clinical significances of angiopoietin-1, -2 and Tie2 in human gastric cancer. Biochem. Biophys. Res. Commun..

[31] Papapetropoulos A., Fulton D., Mahboubi K., Kalb R.G., O'Connor D.S., Li F., Altieri D.C., Sessa W.C. (2000). Angiopoietin-1 inhibits endothelial cell apoptosis via the Akt/survivin pathway. J. Biol. Chem..

[32] Stoeltzing O., Ahmad S.A., Liu W., McCarty M.F., Wey J.S., Parikh A.A., Fan F., Reinmuth N., Kawaguchi M., Bucana C.D., Ellis L.M. (2003). Angiopoietin-1 inhibits vascular permeability, angiogenesis, and growth of hepatic colon cancer tumors. Cancer Res..

[33] Sinan K.I., Dall’acqua S., Ferrarese I., Mollica A., Stefanucci A., Glamočlija J., Sokovic M., Nenadić M., Aktumsek A., Zengin G. (2021). Lc-ms based analysis and biological properties of pseudocedrela kotschyi (Schweinf.) harms extracts: a valuable source of antioxidant, antifungal, and antibacterial compounds. Antioxidants.

[34] Abbasi M., Gholizadeh R., Kasaee S.R., Vaez A., Chelliapan S., Fadhil Al-Qaim F., Deyab I.F., Shafiee M., Zareshahrabadi Z., Amani A.M., Mosleh-Shirazi S., Kamyab H. (2023). An intriguing approach toward antibacterial activity of green synthesized Rutin-templated mesoporous silica nanoparticles decorated with nanosilver. Sci. Rep..

[35] Rauf A., Imran M., Abu-Izneid T., Iahtisham-Ul-Haq, Patel S., Pan X., Naz S., Sanches Silva A., Saeed F., Rasul Suleria H.A., Proanthocyanidins (2019). A comprehensive review. Biomed. Pharmacother..

[36] Rempe C.S., Burris K.P., Lenaghan S.C., Stewart C.N. (2017). The potential of systems biology to discover antibacterial mechanisms of plant phenolics. Front. Microbiol..

[37] Silva A., Silva V., Igrejas G., Gaivão I., Aires A., Klibi N., Dapkevicius M.L.E., Valentão P., Falco V., Poeta P. (2021). Valorization of winemaking by-products as a novel source of antibacterial properties: new strategies to fight antibiotic resistance. Molecules.

